# Vitamin D Intake and Factors Associated With Self-Reported Vitamin D Deficiency Among US Adults: A 2021 Cross-Sectional Study

**DOI:** 10.3389/fnut.2022.899300

**Published:** 2022-05-11

**Authors:** Jeanette M. Andrade, Philip G. Grandoff, Sydney T. Schneider

**Affiliations:** Food Science and Human Nutrition Department, University of Florida, Gainesville, FL, United States

**Keywords:** vitamin D, adults, food frequency questionnaire, chronic conditions, sun exposure

## Abstract

Vitamin D deficiency is a global issue that may be attributed to various factors such as dietary habits, sun exposure, age, race and chronic conditions. The purpose of this study was to determine the relationship between vitamin D intake from food/supplements and factors that may be associated with self-reported vitamin D deficiency among US adults. A cross-sectional online study was conducted among 1,637 adults using a 38-item questionnaire. Frequency counts and percentages were tabulated and a multiple linear regression was performed. Statistical significance was determined at *p* < 0.05. Participants (*n* = 554, 33.8%) were considered vitamin D deficient and consumed an average of 347.05 ± 307.8 IUs of vitamin D through foods/beverages. The multivariate linear regression showed no statistically significant difference with vitamin D intake from foods/beverages on vitamin D deficiency status. Significant positive correlations were seen with vitamin D deficiency status and certain chronic conditions such as chronic kidney disease (*p* = 0.04), depression (*p* < 0.001), diabetes (*p* = 0.02), and vitamin D supplement use (*p* < 0.001). Significant negative correlations were observed with vitamin D deficiency status and age (*p* = 0.01) and sun exposure (*p* < 0.001). Future focus should be on educating individuals about factors associated with vitamin D to reduce the prevalence of vitamin D deficiency.

## Introduction

Vitamin D, a fat-soluble vitamin, regulates serum calcium and phosphate to aid with bone mineralization, reduce inflammation and modulate immune function (1). Vitamin D can be obtained naturally through few food sources such as fatty fish, egg yolks and red meat, fortified foods such as cereals and fortified beverages such as milk, and through sun exposure (2). When vitamin D is obtained through food or sunlight, it undergoes two hydroxylations in the liver and kidney prior to activation. In the liver, vitamin D is converted to calcifediol [25(OH)D] and in the kidney, 25(OH)D-1α-hydroxylase, creates the active form, 1,25(OH)2D (3, 4). A study had illustrated that 5.9% of the US population were considered vitamin D deficient (serum vitamin D concentrations of <30 nmol or 12 ng/mL) and 24% of the US population were considered vitamin D insufficient (serum vitamin D concentrations of <50 nmol or <20 ng/mL) (5). Individuals at highest risk for vitamin D insufficiency/deficiency are those of advanced age, geographical location, race, sunscreen use, and significant time spent indoors (6–9). Furthermore, cross-sectional studies have illustrated that individuals who have certain chronic diseases such as kidney and obesity are at a higher risk for vitamin D deficiency (6, 10).

In the United States, 51.8% of adults have been diagnosed with at least one chronic disease (11) with the most prominent being heart disease, diabetes and kidney disease (12–14). Multiple factors contribute to chronic diseases such as age, sex, lifestyle behaviors such as limited physical activity (<150 min/week) (15) and dietary habits such as high intake of sodium, total fats and low intake of fruits, vegetables, and fiber (14, 16–19). Dietary habits that reduce intake of fruits, vegetables and other nutritious foods and beverages contributes to underconsumption of vitamin D (14, 20). The recommended daily allowances (RDA) of vitamin D for adults, aged 19–69 years, is 600 IUs or 15 mcgs daily and for adults >70 years old is 800 IUs or 20 mcgs daily with a tolerable upper limit of 4,000 IUs (21). Based on the results from the 2015–2016 NHANES dataset, men and women consumed, on average, 201.4 and 168 IU of vitamin D, respectively (22). Even though cross-sectional studies have shown the intake of vitamin D through supplements and dietary intake among American adults, they report this information based on 24-h recalls. Twenty-four-hour recalls can provide more accurate estimations of intake but tend to be labor-intensive and contribute to over or underreporting of information. Additionally, these only give a small insight to the dietary habits of an individual (23, 24). On the other hand, food frequency questionnaires (FFQs) are used for epidemiological studies as they are practical, easy to use and can provide an estimation of nutrients consumed based on the frequency, types and quantities of foods/beverages consumed (25, 26). Various FFQs have been validated to determine vitamin D intake in adults of various age ranges (e.g., young adults, college-aged athletes, post-menopausal females) (7, 27–30). However, one validated FFQ not only assesses vitamin D intake from food, but also sunlight exposure, sunscreen use, and supplement intake (7).

Strategies to improve vitamin D status, dependent on chronic disease or illness, has been to prescribe supplements in various quantities (e.g., 200–540,000 IU), frequencies (e.g., once to monthly) and forms (e.g., vitamin D2 or D3) (31–33). For healthy adults who are vitamin D deficient, 800 IUs daily of vitamin D supplements is recommended to achieve a sufficient serum 25(OH)D level of >20 ng/mL (34). Based on results from a 2015–2016 study, 49% of men and 59% of women aged 60 years and older took a vitamin D supplement, regardless if they were vitamin D deficient (22). Therefore, the purpose of this study was to explore the relationship between frequency and type of vitamin D intake from foods/beverages and risk factors associated with self-reported vitamin D deficiency among US adults.

## Methods

### Study Design and Participants

A cross-sectional study was conducted online through QualtricsXM, an online survey platform, during July–September 2021. Recruitment was voluntary and anonymous and occurred through e-blasts, social media platforms and ResearchMatch. ResearchMatch is a national health volunteer registry created by several academic institutions and supported by the National Institutes of Health as part of the Clinical Translational Science Award (CTSA) program (35). Inclusion criterion was adults being over the age of 18 years. Individuals who were not 18 years of age were excluded from the study. Initially a total of 1,837 adults consented to the study with 1,637 participants having complete data. All aspects of the study were approved exempt by the Institutional Review Board at the University of Florida (IRB202100807).

### Questionnaire

Participants responded to a 38-item validated vitamin D questionnaire (7) that included demographics (10 items), sunlight exposure (4 items), supplement use (5 items), and foods/beverages consumed (24 items). Demographic questions included age range, sex, race/ethnicity, state of residence for the past 11 months, vitamin D insufficient/deficient, and chronic diseases/conditions. The vitamin D insufficient/deficient question had participants select yes/no or unsure, if participants selected yes, another question appeared with when they were diagnosed. Multiple selections were provided for the chronic diseases/conditions, if participants selected chronic kidney/renal disease, three additional prompt questions appeared that focused on when they were diagnosed, the stage they are currently in and stage they were in when initially diagnosed.

For questions surrounding sunlight exposure, participants selected the length of time they generally spent in the sun. Based on state of residence, participants were categorized as either receiving low, medium, or high sun exposure based on the National Environmental Public Health tracking network (36). Participants also identified the use of sunscreen as never/rarely, sometimes, usually or always. If they responded sometimes, usually or always, they received an additional question about the sunscreen SPF they used. For supplement use, if participants selected anything else but never, a prompt appeared for them to provide a free text response about the dosage.

The frequency of consuming foods/beverages that contained vitamin D either naturally or fortified were slightly modified from the original questionnaire to include additional sources of vitamin D foods (e.g., cheeses) and removal of sub-way sandwich (see Supplementary Table 1). The modified questionnaire included a total of 24 food/beverage items that provided portion sizes and categorized based on food group. Participants selected the frequency of consuming these foods/beverages over a 30-day period from never, rarely, 1–2 portions/week, 3–5 portions/week, one portion/day, 2–3 portions/day, and 4 or more portions/day. Adhering to the methodology by Larson-Meyer et al. (7), the intake of vitamin D from foods and beverages was estimated by multiplying the frequency midpoint by the average content of vitamin D and subsequently expressed as International Units (IU) daily over the 30-day period.

### Statistical Analysis

Frequency counts and percentages were tabulated for demographic variables, average vitamin D intake, frequency of vitamin D supplement intake, and exposure to sun. Multiple linear regression was conducted to examine the relationships between self-reported vitamin D status and vitamin D intake from foods/beverages, supplements, sun exposure, and demographics (sex, race, age, chronic conditions/diseases, and state of residence). This analysis examined the key study co-variates (vitamin D intake from foods/beverages, supplements, and chronic conditions/diseases) and confounding variables (sex, race, age, state of residence, sun exposure) and isolated the relationship of interest—vitamin D status. Statistical significance was determined at *p* < 0.05 using JMP SAS v16 (SAS institute Inc, Cary, NC).

## Results

### Study Population

A total of 1,637 adults with a total completed survey were included in the analysis. A majority of participants were female (74.8%), between the ages of 30–49 years (27.1%), who identified as white/ non-Hispanic (86.3%), were never informed they were vitamin D deficient (60.0%), regardless of vitamin D status, 36.6% took a vitamin D supplement at least once daily, and a majority lived in a state that was considered low UV exposure (46.4%). Few participants indicated that they had a chronic disease/condition. Participants spent between 0.5–1 and 1–2 h in the sun daily from 10 a.m. to 3 p.m. (19.0 and 19.5%, respectively), and sometimes wore sunscreen (35.8%) and if they wore sunscreen chose an SPF of 50+ (36.1%) (see Table 1).

**Table 1:** Participants' Demographics (*n* = 1,637).

**Variables**	**Number (%)**
**Sex**	
Female	1,225 (74.8%)
Male	381 (23.3%)
Not reported	24 (1.5%)
**Age**	
18–24	80 (4.9%)
25–29	116 (7.1%)
30–49	443 (27.1%)
50–59	282 (17.2%)
60–69	391 (23.9%)
70+	310 (18.9%)
Not reported	1 (0.8%)
**Race/Ethnicity**	
African American	64 (3.9%)
Asian	30 (1.8%)
Hispanic	39 (2.4%)
Native American	8 (0.5%)
White	1,412 (86.3%)
Other	55 (2.4%)
Not reported	26 (1.6%)
**Sun exposure**	
None	75 (1.3%)
1–3 h per month	115 (7.0%)
1 h per week	159 (9.7%)
2–4 h per week	298 (18.2%)
5–6 h per week	184 (11.2%)
1 h per day	311 (19.0%)
1–2 h per day	320 (19.5%)
2 or more hours per day	166 (10.1%)
Not reported	8 (0.5%)
**Sunscreen use**	
Never	389 (23.8%)
Sometimes	586 (35.8%)
Usually	428 (26.1%)
Always	226 (13.8%)
Not reported	3 (0.2%)
**Sunscreen SPF amount**	
None	454 (27.7%)
1–14	7 (0.4%)
15–29	66 (4.0%)
30–49	518 (31.6%)
50+	591 (36.1%)
**State of Residence**	
Low UV exposure	759 (46.4%)
Medium UV exposure	433 (26.5%)
High UV exposure	367 (22.4%)
Not reported	77 (4.7%)
**Vitamin D status**	
Not deficient	981 (59.9%)
Deficient	554 (33.8%)
Unknown	101 (6.2%)
**Chronic conditions/diseases**	
Cancer	98 (6%)
Depression	437 (26.7%)
Diabetes	155 (9.5%)
Diverticulosis/Diverticulitis	87 (5.3%)
Gastric reflux	291 (17.8%)
Heart disease	323 (19.7%)
Irritable bowel	189 (11.6%)
Liver disease	41 (2.5%)
Lung disease	69 (4.2%)
Chronic kidney disease (CKD)	39 (2.4%)
**Frequency of taking vitamin D supplements**	
None	705 (43.1%)
1–2 times per week	103 (6.3%)
3–5 times per week	93 (5.7%)
Once per day	598 (36.6%)
2 or more times per day	80 (4.9%)
Not reported	57 (3.5%)

### Vitamin D Intake From Foods and Beverages

On average, participants consumed 347.05 ± 307.8 IUs of vitamin D from foods/beverages. Foods/beverages that contained vitamin D were never or rarely consumed except for milk and milk products that were consumed once daily (20.7%), chicken that was consumed 3–5 times per week (37.7%), and orange juice or other juices fortified with vitamin D (26.4%), cheddar or hard cheeses (33.2%), entire egg (33.5%), and beef/pork (39.5%) consumed 1–2 times per week (see Table 2).

**Table 2:** Percent frequency of consuming vitamin D in foods and beverages (*n* = 1,637).

	**4 portions or more per day**	**2–3 portions per day**	**1 portion per day**	**3–5 portions per week**	**1–2 portions per week**	**Rarely**	**Never**
Salmon, rainbow trout, herring, eel	0.1	0.2	0.1	3.4	31.3	31.0	33.9
Halibut, mackerel, brook trout, sole, tuna	0	0.2	0.3	1.6	20.1	39.5	38.2
Cod, flounder, pollock, bass	0	0.2	0.1	1.0	11.6	37.6	49.4
Tilapia	0	0.1	0.1	0.5	7.9	29.3	62.0
Herring, sardine, tuna	0.1	0.1	0.2	1.5	21.2	35.9	41.1
Other fish products	0.2	0.1	0.7	1.0	12.3	38.2	47.5
Mushrooms, white, raw	0.8	1.5	1.8	6.1	25.4	31.0	33.3
Milk and milk beverages (yogurt, buttermilk)	2.9	17.6	20.7	16.6	16.2	14.5	11.4
Soy or alternative milks fortified with vitamin D	0.7	3.2	9.9	7.3	10.0	18.1	50.7
Blue or soft cheeses	0.1	0.9	2.6	7.3	26.0	34.5	28.5
Cheddar or hard cheese	0.7	5.0	13.4	24.6	33.2	13.1	10.0
Cottage cheese	0	0.7	4.0	10.8	30.8	32.9	20.5
Ice cream (dairy)	0.2	0.7	4.0	10.8	30.8	32.9	20.5
Entire egg	0.8	6.4	10.9	24.9	33.5	14.2	9.2
Egg yolk, plain no white	0.1	0.7	1.3	2.2	5.3	20.0	70.4
Beef, pork	0.5	1.8	4.9	21.5	39.5	16.4	15.3
Chicken	0.5	3.4	8.4	37.7	33.2	5.6	11.1
Beef liver	0.1	0.1	0.2	0.3	0.7	10.9	87.7
Hot dogs, processed meats, sausage, bacon	0	0.8	2.1	7.0	30.2	34.3	25.5
Cereals fortified	0.5	1.7	8.4	9.5	18.3	20.6	40.9
Orange juices or others fortified	0.7	7.3	11.9	21.0	26.4	17.4	15.2
Margarine	0.4	1.3	6.6	8.0	16.6	30.2	36.8
Butter, butter products	0.1	2.0	3.8	6.0	9.2	18.2	60.7

### Relationship Between Vitamin D Intake, Risk Factors on Self-Reported Vitamin D Deficiency

The multivariate linear regression showed significant positive correlations for chronic kidney disease (0.05, CI 95% 0.01–0.40; *p* = 0.04), depression (0.07, CI 95% 0.03–0.17; *p* < 0.001), diabetes (0.06, CI 95% 0.02–0.23; *p* = 0.02), and vitamin D supplement use (0.17, CI 95% 0.05–0.08; *p* < 0.001) on vitamin D status. Significant negative correlations were discovered with age (−0.08, CI 95% −0.06 to −0.01; *p* = 0.01) and sun exposure (−0.09, CI 95% −0.04−0.01; *p* < 0.001) on vitamin D status (see Table 3).

**Table 3:** Multivariable associations on vitamin D status.

**Variables**	**Coef**.	**Std. err**.	***t***	***P* > |t|**	**[95% conf. interval]**	
Sex	0.04	0.01	1.37	0.17	−0.01	0.05
Race	0.03	0.01	0.99	0.32	−0.1	0.04
Age	−0.08	0.01	−2.81	0.01*	−0.06	−0.01
Cancer	0.02	0.06	0.65	0.52	−0.08	0.17
Chronic kidney disease	0.05	0.10	2.07	0.04*	0.01	0.40
Depression	0.07	0.04	2.86	<0.001*	0.03	0.17
Diabetes	0.06	0.05	2.32	0.02*	0.02	0.23
Diverticulosis/diverticulitis	0.03	0.07	1.15	0.25	−0.06	0.21
Gastric reflux	0.07	0.04	2.78	0.01	0.03	0.20
Heart Disease	0.01	0.04	0.45	0.65	−0.06	0.10
Irritable bowel	0.01	0.05	0.23	0.82	−0.08	0.11
Liver disease	−0.01	0.10	−0.54	0.59	−0.24	0.14
Lung disease	0.03	0.08	0.99	0.33	−0.08	0.23
State of residence	0.01	0.02	0.26	0.80	−0.03	0.04
Sun exposure	−0.09	0.01	−3.70	<0.001*	−0.04	−0.01
Sunscreen use	0.02	0.02	0.86	0.39	−0.02	0.04
Vitamin D supplements	0.17	0.01	6.52	<0.001*	0.05	0.08
Vitamin D intake from foods/beverages	−0.03	0.00	−1.03	0.30	0.00	0.00

*p < 0.05.

## Discussion

Vitamin D deficiency is prevalent within the United States. Findings from this study demonstrated that 38.8% of participants had indicated they were vitamin D deficient and that associations were observed regarding vitamin D status with age, sun exposure, vitamin D supplement intake, and conditions.

Average total IUs of vitamin D intake daily from this study was 347.05 ± 307.8, which was similar to the intake reported by another study (7). Larson-Meyer et al. (7) demonstrated that participants' average consumption of vitamin D from foods and beverages was between 341 ± 228 IUs (*n* = 86) and 584 ± 589 IUs (*n* = 49), dependent on the season; fall, winter or spring. Although, the findings from this study showed that reported average vitamin D consumption was higher from foods and beverages compared to other studies that had reported vitamin D consumption between 68 IUs (37) and 178 IUs (30). This discrepancy may exist due to the different instruments used to obtain vitamin D intake. In this study, a modified validated food frequency questionnaire was used to determine the frequency participants consumed foods that contained vitamin D over a 30-day period. In the other studies (30, 37), 24-h recalls were used to determine amount of vitamin D consumed. Other studies demonstrated a discrepancy in vitamin D intake averages from food frequency questionnaires and 24-h recalls from 3.2% (38)−84% (30) likely due to over/under estimation of reporting or inability to recall frequency of consuming foods/beverages over certain time (39, 40). Overall, though, participants, on average, did not meet the vitamin D RDAs for healthy people. This is mainly related to participants rarely or never consuming foods high in vitamin D such as fish and dairy products. On the other hand, regardless of vitamin D status, 53% of participants took a vitamin D supplement at least once per week with the majority (36.6%) taking a vitamin D supplement at least once daily. An attempt was to gather the IUs/mcgs that participants took of vitamin D supplements, but many did not complete this section nor completed this correctly (e.g., provided the amount without indication of the units or provided amounts that were not commonly found in vitamin D supplements−600 mgs), thus no further analysis was able to be performed with how the supplement amount accounted for the total vitamin D intake.

Participants who completed this study may have been considered healthy as few indicated they had been diagnosed with the chronic conditions/diseases that have been associated with vitamin D deficiency. Although, positive associations were found with vitamin D status and chronic condition/disease in which those who had indicated they were depressed, diagnosed with diabetes, GI conditions such as gastric reflux, and chronic kidney disease had identified they were vitamin D deficient. This corresponds to other studies that showed associations between lower serum 25(OH)D levels (e.g., <20 ng/mL or 50 nmol/L) and the above conditions (41–44).

Furthermore, participants who had indicated sun exposure of 30 min or more daily had indicated no vitamin D deficiency. On the other hand, there were no associations between place of residence (low, middle or high UV exposure) and vitamin D status. This is in opposition to studies that have suggested that individuals who live in areas where there is low UV exposure, they are at a higher risk for vitamin D deficiency (6, 10, 45, 46). There was a negative association between age and vitamin D status, in which younger individuals had identified they were vitamin D deficient. Marzban et al. (47) observed that participants (*n* = 1,806) who were between the ages of 30–39 had lower serum vitamin D levels compared to participants who were 80 years and older. This was further confirmed by analyses of NHANES data (*n* = 25,010) that showed 42.4% of individuals between the ages of 18–39 years old were vitamin D deficient compared to 21.0% of individuals aged 60 years and older (48). Younger individuals may be at a higher risk for vitamin D deficiency due to low consumption of foods and beverages naturally high in vitamin D or limited sun exposure (49, 50). As this study was conducted over the summer for most of the United States, it is unknown if participants obtain the same sun exposure during colder months. Moreover, few, 13.7% of participants, met the RDAs for vitamin D, which could explain the number of participants who were <60 years of age indicating they were vitamin D deficient. Another explanation could be that participants who were older were taking a vitamin D supplement compared to the younger participants. There were no associations between sunscreen use and vitamin D deficiency, which is similar to findings from other studies (51, 52).

Limitations of this study included self-reporting of dietary intake and vitamin D status level. Further studies should focus on obtaining vitamin D serum samples and collecting them in different seasons. Even though participants from across the United States participated in this study, limited generalization of the results can occur from this study due to the limited variability of the demographics. As this study was conducted online, individuals who were without internet or were limited in their technological capability may have been excluded from participating (53, 54). Furthermore, this study was conducted while COVID-19 was occurring, thus ability to purchase seafood or other high vitamin D foods due to various reasons may have been limited, so conducting a follow-up study would be necessary for identifying shifts in consumption patterns.

## Conclusion

In conclusion, this study showed that through self-reporting, the prevalence of vitamin D deficiency was nearly 39%. Foods that are naturally rich in vitamin D were rarely/never consumed. Although, intake of vitamin D supplements occurred frequently. Associations were seen with certain chronic conditions/diseases and vitamin D deficiency. Therefore, attempts should be made among the educational and scientific community to develop nutrition education programs to educate individuals about consuming and preparing foods naturally high in vitamin D to reduce the prevalence of vitamin D deficiency.

## Data Availability Statement

The original contributions presented in the study are included in the article/Supplementary Material, further inquiries can be directed to the corresponding author/s.

## Ethics Statement

The studies involving human participants were reviewed and approved by University of Florida Institutional Review Board. The patients/participants provided their written informed consent to participate in this study.

## Author Contributions

JA: conceptualization of this study. JA, PG, and SS: methodology, formal analysis, investigation, and writing—review and editing. JA: data curation, writing—original draft preparation, supervision, and project administration. All authors have read and agreed to the published version of the manuscript.

## Conflict of Interest

The authors declare that the research was conducted in the absence of any commercial or financial relationships that could be construed as a potential conflict of interest.

## Publisher's Note

All claims expressed in this article are solely those of the authors and do not necessarily represent those of their affiliated organizations, or those of the publisher, the editors and the reviewers. Any product that may be evaluated in this article, or claim that may be made by its manufacturer, is not guaranteed or endorsed by the publisher.
