# Supplementary material for: Vitamin D Intake and Factors Associated With Self-Reported Vitamin D Deficiency Among US Adults: A 2021 Cross-Sectional Study
Source: Front Nutr. 2022 May 11;9:899300. doi: 10.3389/fnut.2022.899300 (PMC9131078; doi:10.3389/fnut.2022.899300)
Supplement: Supplementary file 1 [file Data_Sheet_1.docx]

**Supplemental Table 1:**

**Instrument: Vitamin D Questionnaire**

**For the past month, indicate how often you consumed the following portions of foods or beverages**

Use the following portion sizes to estimate how many portions you eat/drink:

Fish, meat, chicken: 3 ounces cooked

Vegetable: ½ cup raw

Dairy: Milk = 1 cup (8 ounces); Cheese 1 ounce/1 slice; Cottage cheese/ice cream: ½ cup

Eggs: 1 large

Grains (cereal, pasta, bread): 1 cup, ½ cup cooked, 1 slice of bread

Beverages: Juices = ½ cup (4 ounces)

Fats: 1 tsp.

| **Group of products** | **Products** | **4 or more portions per day** | **2-3 portions per day** | **One portion per day** | **3-5**  **portions/ week** | **1-2**  **portions/ week** | **Rarely** | **Never** |
| --- | --- | --- | --- | --- | --- | --- | --- | --- |
| Fresh and Smoked Fish | Salmon, rainbow trout, herring, eel |  |  |  |  |  |  |  |
|  | Halibut, mackerel, brook trout, sole, tuna |  |  |  |  |  |  |  |
|  | Cod, flounder, pollock, bass |  |  |  |  |  |  |  |
|  | Tilapia |  |  |  |  |  |  |  |
| Fish | Herring, sardine, tuna |  |  |  |  |  |  |  |
|  | Other fish products |  |  |  |  |  |  |  |
| Vegetable | Mushrooms, white, raw |  |  |  |  |  |  |  |
| Dairy | Milk and milk beverages (yoghurt, buttermilk) |  |  |  |  |  |  |  |
|  | Soy or alternative milks fortified with vitamin D |  |  |  |  |  |  |  |
|  | Blue or soft cheeses |  |  |  |  |  |  |  |
|  | Cheddar or hard cheese |  |  |  |  |  |  |  |
|  | Cottage cheese |  |  |  |  |  |  |  |
|  | Ice cream (dairy) |  |  |  |  |  |  |  |
| Eggs | Entire egg |  |  |  |  |  |  |  |
|  | Egg yolk, plain no white |  |  |  |  |  |  |  |
| Meat | Beef, pork |  |  |  |  |  |  |  |
|  | Chicken |  |  |  |  |  |  |  |
|  | Beef liver |  |  |  |  |  |  |  |
| Meat products | Hot dogs, processed meats, sausage, bacon |  |  |  |  |  |  |  |
| Grains | Corn flakes, Raisin bran, cheerios – others fortified with vitamin D |  |  |  |  |  |  |  |
|  | Breads, pastas fortified with vitamin D |  |  |  |  |  |  |  |
| Beverage | Orange or others fortified with vitamin D |  |  |  |  |  |  |  |
| Fats | Margarine |  |  |  |  |  |  |  |
|  | Butter, butter products |  |  |  |  |  |  |  |

***Supplement Use***

For each supplement, mark the box indicating how often you take this supplement over the past month

| **Vitamins** | **4 or more times per day** | **2-3 times per day** | **One time per day** | **3-5**  **times/ week** | **1-2**  **times/ week** | **Rarely** | **Never** |
| --- | --- | --- | --- | --- | --- | --- | --- |
| Multiple |  |  |  |  |  |  |  |
| Calcium |  |  |  |  |  |  |  |
| Calcium + vitamin D |  |  |  |  |  |  |  |
| Vitamin D |  |  |  |  |  |  |  |

Based on the supplements above that you take, indicate the dosage. (For example, 500mg of calcium)

***Sunlight Exposure***

Over the past month, how much leisure time is spent out in the sun between 10am-3pm

1. >2 hours per day
2. 1-2 hours per day
3. 1/2 – 1 hour per day
4. 5-6 hours per week
5. 2-4 hours per week
6. 1 hour per week
7. 1-3 hours per month
8. Never or <1 hour per month

When you are outside, how often do you use sunscreen?

1. Always
2. Usually
3. Sometimes
4. Never

What sunscreen SPF do you use?

Over the past month, when you go outside, what do you typically wear (mark all that apply)

1. Long sleeve shirt
2. Short sleeve shirt
3. Swimsuit/trunks
4. Shorts
5. Pants
6. Hat (baseball, other)
7. Gloves (gardening, winter)
8. Other
